# Brain alteration of PCOS: neuroimaging and function

**DOI:** 10.3389/fneur.2026.1825907

**Published:** 2026-06-11

**Authors:** Ningxiao Jiang, Jie Deng, Changjin Bao, Hongmei Yin, Xianghui Zhang, Yanxia Ding, Shinan Zhang, Yingjiang Xu, Xinghua Diao, Kexin Lu, Jun Liu, Lei Han

**Affiliations:** 1 Department of Reproductive Medicine, Shandong Medical and Pharmaceutical University Hospital, Shandong Medical and Pharmaceutical University, Binzhou, Shandong Province, China; 2 Department of Gynecology, The First Affiliated Hospital of Traditional Chinese Medicine of Chengdu Medical College, XinDu Hospital of Traditional Chinese Medicine, Chengdu, Sichuan Province, China; 3 Department of Radiology, Shandong Medical and Pharmaceutical University Hospital, Shandong Medical and Pharmaceutical University, Binzhou, Shandong Province, China; 4 Department of Interventional Vascular Surgery, Shandong Medical and Pharmaceutical University Hospital, Shandong Medical and Pharmaceutical University, Binzhou, Shandong Province, China; 5 Department of Obstetrics and Gynecology, Shandong Medical and Pharmaceutical University Hospital, Shandong Medical and Pharmaceutical University, Binzhou, Shandong Province, China; 6 Department of Gynecology, Hainan General Hospital, Hainan Affiliated Hospital of Hainan Medical University, Haikou, Hainan Province, China

**Keywords:** brain alteration, cerebral functional, endocrine diseases, neuroimaging, polycystic ovary syndrome

## Abstract

Polycystic ovary syndrome (PCOS), a common endocrine disorder affecting women, continues to be inadequately understood in terms of its pathogenesis. However, it is strongly associated with structural and functional abnormalities in the brain. This review examines neuroimaging alterations and cerebral functional changes observed in patients with PCOS, investigating their relationships with endocrine-metabolic profiles, behavioral patterns, cognitive performance, and emotional regulation. Neuroimaging studies have identified several key findings, including the enlargement of the pituitary gland (encompassing both the anterior and posterior regions, but excluding the infundibular stalk), a reduction in gray matter volume, and damage to the corpus callosum within the white matter. In patients with PCOS, structural alterations and changes in activity states within specific brain regions are associated with abnormal glucose metabolism, disordered eating behaviors, and cognitive and emotional changes. Notably, elevated μ-opioid receptor binding capacity in emotion-related brain regions and altered activity in the right orbitofrontal cortex under sympathetic activation has been observed. Collectively, these alterations point to a dysfunction of the limbic system circuitry, which may underlie the prevalent affective and cognitive symptoms in PCOS. Such neural imaging changes provide critical insights into PCOS pathophysiology and therapeutic development, though further investigations are warranted to address unresolved mechanistic questions.

## 1 Introduction

Polycystic ovary syndrome (PCOS) is a prevalent endocrine disorder in women of reproductive age, characterized by irregular menstruation, hyperandrogenemia and/or clinical manifestations of hyperandrogenism, and polycystic ovarian morphology. Emerging evidence suggests that its clinical manifestations are closely associated with neuroendocrine dysregulation involving both the cerebral cortex and the hypothalamic-pituitary-ovarian (HPO) axis (1–4). The underlying pathogenesis of PCOS remains

incompletely understood; it is postulated to involve a complex interplay of insulin resistance (IR), aberrant hormonal profiles, and persistent chronic inflammatory stimuli. Cortical dysfunction may disrupt the HPO axis, exacerbating endocrine dysregulation, and vice versa, although the correlates of specific brain regions with clinical manifestations alterations remain uncharacterized (2). Beyond the cerebral cortex, the hypothalamus and pituitary gland represent critical research foci in PCOS. Their morphological alterations and secretory dysfunctions may trigger ovarian theca cell hyperplasia, excessive androgen production, and polycystic ovarian remodeling, collectively driving the pathogenesis of PCOS (5). However, current investigations remain limited in scope, with somewhat controversial across studies complicating the interpretation of neural correlates (6, 7). This study systematically synthesizes imaging evidence to delineate cerebral cortical, limbic (e.g., amygdala, hippocampus), hypothalamic, and pituitary alterations in PCOS patients, with particular emphasis on their correlations with metabolic dysregulation, behavioral phenotypes (including dietary behaviors and cognitive performance), affective states, and laboratory biomarkers. By elucidating these neuroendocrine-metabolic-behavioral interrelationships, this review aims to inform the development of biomarker-driven diagnostic criteria and targeted therapeutic strategies aimed at optimizing clinical outcomes and health-related quality of life in affected individuals.

## 2 Methods

This systematic review was conducted in accordance with the Preferred Reporting Items for Systematic Reviews and Meta-Analyses (PRISMA) statement to ensure methodological rigor and transparency.

The literature search was designed to comprehensively capture studies investigating cerebral structural and functional alterations in patients with PCOS. Both medical subject headings (MeSH terms) and free-text terms were used to construct the search strategy, with logical combinations of three core concepts: polycystic ovary syndrome (PCOS) AND neuroimaging/ cerebral structure/ cerebral function AND endocrine metabolism/ cognition/ emotion/ sympathetic activation. Key Search terms included: “polycystic ovary syndrome,” “PCOS,” “brain,” “cerebrum,” “neuroimaging,” “magnetic resonance imaging (MRI),” “diffusion tensor imaging (DTI),” “positron emission tomography (PET),” “functional MRI (fMRI),” “gray matter volume,” “white matter integrity,” “pituitary gland,” “hypothalamus,” “glucose metabolism,” “cognition,” “emotion regulation,” “sympathetic nervous system,” “μ-opioid receptor.” PubMed and Web of Science were searched without geographical or initial time restrictions to maximize retrieval of relevant studies.

For the inclusion criteria, this review included original studies (cross-sectional studies, case-control studies, cohort studies) and systematic reviews/meta-analyses focusing on PCOS-related cerebral alterations. The study participants were women with PCOS diagnosed according to the Rotterdam criteria, National Institutes of Health (NIH) criteria, or Androgen Excess Society (AES) criteria, as well as age- and body mass index (BMI)-matched healthy female controls. Eligible studies were required to report outcomes related to cerebral structure (e.g., gray matter volume, pituitary gland volume, white matter integrity) or cerebral function [e.g., glucose metabolic rate, amplitude of low-frequency fluctuation (ALFF), functional connectivity (FC)], along with correlations between these outcomes and clinical indicators such as endocrine metabolism (insulin resistance, hormone levels), cognition, and emotion; only English-language publications were included. The exclusion criteria comprised duplicate publications (only the most comprehensive or latest version was retained), gray literature (conference abstracts, letters to the editor, case reports with a sample size < 5, unpublished theses, and commentaries), studies with incomplete data, unextractable key outcomes, or severe methodological flaws (e.g., lack of a control group, unvalidated PCOS diagnostic criteria), studies focusing on non-neuroimaging outcomes (e.g., only investigating ovarian or metabolic abnormalities in PCOS), studies involving patients with comorbid neurological diseases (e.g., Alzheimer's disease, multiple sclerosis) or severe mental disorders (e.g., schizophrenia) and studies if participants were undergoing specific pharmacological treatments for PCOS (e.g., insulin sensitizers, ovulation induction agents) or using hormonal contraceptives.

Literature screening was conducted in three stages by two independent reviewers (Ningxiao Jiang and Shinan Zhang): Firstly, duplicate records across databases were removed using EndNote X9 software; secondly, studies that did not meet the basic inclusion criteria (e.g., non-PCOS population, absence of any neuroimaging data) were excluded based on their titles and abstracts; finally, full texts of the remaining studies were retrieved to confirm eligibility against the final inclusion/exclusion criteria. Disagreements between the two reviewers at any stage were resolved through discussion, and if consensus could not be reached after discussion, a third independent reviewer (Lei Han) was consulted for arbitration. Additionally, supplementary literature was identified through manual searches of the reference lists of included studies, key review articles, and relevant meta-analyses, with duplicate and ineligible studies excluded from this process.

**Figure 1:**
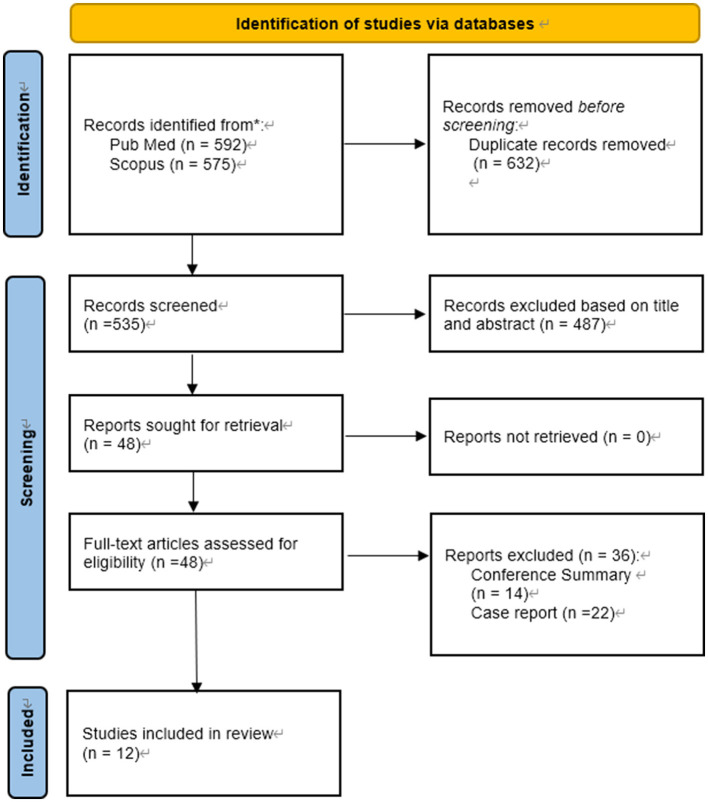
PRISMA flow diagram.

A total of 1,167 articles were identified through the initial literature search. After removing duplicates *(n* = 632), 487 studies were then excluded by screening titles and abstracts because they did not fulfill the inclusion criteria. The full text of the remaining 48 potentially relevant studies was examined, of which only 12 met the eligibility criteria for inclusion in this systematic review. The selection process is shown in Figure 1 (Table 1).

**Table 1:** Key characteristics of samples in the main studies.

References	Imaging modality	Year spanned	Total sample size	Sample size	Diagnostic criteria	Drug administration	Mean age	BMI (kg/m^2^)	Brain region alterations (Key findings)	Race/ethnicity
Unlu et al. (14)	MRI	Year 2014 (January —May)	57	PCOS 26 (Normal weight) (age match)	2003 Rotterdam	none	PCOS 25 ± 2.9	PCOS 22.3 ± 2.7	Pituitary enlarged volume.	none
				Con 31			con 22 ± 3.7	con 23.1 ± 1.7		
Bozkurt et al. (15)	MRI	2016 May−2017 January	81	PCOS 39	The 2003 Rotterdam criteria	Exogenous estrogen (oral contraceptive)	PCOS 24.25 ± 5.5	PCOS 23.06 ± 1.9	Pituitary enlarged volume.	Turkey, Mugla, Sitki, Kocman
				Con 42			con 26.7 ± 5.1			
Ozgen Saydam et al. (12)	MRI	none	40	lean PCOS 10	Androgen excess and the 2003 Rotterdam criteria	none	lean PCOS 20	lean PCOS 21	Obese PCOS Reduced total and regional (thalamus, caudate, hippocampus) gray matter volume Lean PCOS Reduced left amygdala volume	none
				lean con 10			lean con 26.5	lean con 20.5		
				obese PCOS 10			obese PCOS 28.5	obese PCOS 32.4		
				obese con 10			obese con 32	obese con 38.2		
Rees et al. (46)	MRI	none	36	PCOS 18	the 2003 Rotterdam criteria	take oral contraceptive	PCOS 31 ± 6	PCOS30 ± 6	White matter Altered microstructure in corpus callosum/anterior WM	PCOS (18) 16 white (Caucasian)?2 Afro-Caribbean
				Con 18			con 31 ± 7	con 29 ± 6			con (18) all white (Caucasian)
Castellano et al. (27)	PET/MRI	none	18	PCOS 7 (Normal weight, young, mild IR) (age Education match)	the 2003 Rotterdam criteria	oral contraceptive (PCOS + con)	con 24 ± 3.3	normal weight	Gray matter Reduced volume in left superior frontal, supramarginal, right superior parietal gyri.	none	
				Con 11			pcos 24.6 ± 5.9			
Alsaadi HM et al. (72)	BOLD/fMRI	none	19 (All were PCOS patients)	IS 8	the 2003 Rotterdam criteria	oral contraceptive; Antidiabetic drugs	IS 27.3 ± 4.65	IS 30.8 ± 7.03	Limbic system Activity negatively correlated with insulin sensitivity (brain insulin resistance)	none
				IR 11			IR 27.9 ± 5.84	IR 40.0 ± 8.78		
Soleman et al. (100)	fMRI	none	34	PCOS 14	Hyperandrogenism and the 2003 Rotterdam criteria	Antiandrogen therapy	PCOS 29.3 ± 5.6	PCOS25.8 (5.6) 16.9–40.5	Emotion processing Hyperactivation in ventral ACC and left PFC to negative stimuli	none
				Con 20			con 25.6 ± 6.2	con 24.5 (3.0) 20.7–31.1		
Li et al. (22)	rs-fMRI/ ALFF analyse/FC	none	82	PCOS41	the 2003 Rotterdam criteria	Antiandrogen therapy Antidiabetic drugs	PCOS 25.29 ± 3.15	PCOS 24.62 ± 4.88	Sympathetic challenge Altered activity in right OFC	none
				Con 41			con 26.22 ± 2.59	con 20.31 ± 1.81		
Unlu et al. (80)	DWI/ ADC analyse/ MRI/ROI analyse	none	55	PCOS 20	the 2003 Rotterdam criteria	oral contraceptive Antiandrogen therapy Antidiabetic drugs	PCOS 24 ± 1.5	PCOS 23.2 ± 1.1	Corpus Callosum/Anterior WM Elevated ADC, suggesting microstructural alterations.	none
				Con 35			con 25 ± 2.9	con 24.1 ± 2.9		
Guoqing et al. (52)	MR/WMLs assessment/ SCI assessment	none	210	PCOS 70	the 2003 Rotterdam criteria	Antidiabetic drugs	PCOS 55.04 ± 2.55	PCOS 25.44 ± 2.19	Postmenopausal Increased WMLs and silent cerebral infarcts.	none
				Con 140			con 55.11 ± 2.66	con 24.65 ± 2.27		
Lansdown et al. (109)	BOLD fMRI/T1-weighted structural MRI	none	40	PCOS 20(age & BMI matched)	the 2003 Rotterdam criteria	none	PCOS 29.8± 4.8	PCOS 26.05 ± 4.90	Sympathetic challenge Altered activity in right OFC.	none
				Con 20(age & BMI matched)			Con 29.65 ± 4.96	Con 26.11 ± 4.83		
Marsh et al. (38)	fMRI/PET	2008 January - 2009	12	PCOS 7 (IR-PCOS)	the NIH criteria	metformin (PCOS only)	PCOS 26.1 ± 3.5	PCOS 35.3 ± 8.1	μ-opioid system Elevated receptor availability in right amygdala and left NAc.	none
				Con 5 (non-IR controls)			con 26 ± 4	con 23.0 ± 1.5		

## 3 Brain imaging characteristics in patients with PCOS

### 3.1 Pituitary gland imaging alterations in patients with PCOS

The pituitary gland serves as a critical regulator within the female reproductive system. Functioning as the master endocrine gland, it orchestrates the precise developmental and physiological homeostasis of reproductive processes through the secretion of gonadotropins, such as follicle-stimulating hormone (FSH) and luteinizing hormone (LH). This neuroendocrine axis ensures the fidelity of cyclical reproductive events. Patients with PCOS demonstrate dysregulation of the HPO axis, characterized by elevated levels of LH secreted from the pituitary gland and an increased LH/FSH ratio (3, 5). Existing evidence indicates a negative correlation between pituitary gland volume and age, with volumetric enlargement during puberty, pregnancy, and following exogenous estrogen administration (8–11). However, pituitary morphological alterations in PCOS patients remain poorly characterized (12).

A study enrolled normal-weight PCOS patients and body mass index (BMI)-matched healthy controls. All participants underwent MRI scans with T1-weighted sequences during days 2–5 of the menstrual cycle, while basal hormone levels, including LH and the LH/FSH ratio, were measured simultaneously. Pituitary volume was quantified in accordance with established consensus guidelines, which excluded the inferior sphenoid sinus, posterior pituitary, and bilateral infundibulocavernous sinuses during delineation (13). The results demonstrated that the mean pituitary volume in the PCOS group (667.8 ± 55.7 mm3) was significantly larger than that in the control group (525.3 ± 77.8 mm3, *P* < 0.05). Additionally, serum LH levels and LH/FSH ratios were also markedly elevated in the PCOS cohort. Furthermore, pituitary volume exhibited positive correlations with LH levels in both PCOS patients and the overall study population. Multiple linear regression analysis identified the LH/FSH ratio as the sole independent predictor of pituitary volume (*P* = 0.047) (14). A recent study corroborated these findings, reporting significantly larger mean pituitary gland volume (PGV) in PCOS patients compared to controls, along with significant intergroup differences in LH, FSH, and LH/FSH ratios. However, no correlations were observed between PGV and these hormonal parameters in that study (15). In conclusion, two studies on the pituitary gland in PCOS patients have consistently confirmed pituitary enlargement and abnormal hormone levels. However, the underlying mechanisms for this pituitary volume expansion remain unclear, and discrepancies have been observed in its correlation with specific hormone levels. The pituitary enlargement in PCOS patients might represent a non-pathological hypertrophy resulting from functional hyperactivity. Dysfunction of the HPO axis in PCOS may contribute to an accelerated pulsatile secretion rhythm of GnRH (16, 17). This aberrant GnRH pulsatility promotes hypersecretion of LH from the pituitary gland, characterized by elevated baseline levels and heightened pulse frequency (18, 19). The consequent functional activation of pituitary cells, under such prolonged stimulation, may induce morphological alterations within the pituitary gland (20).

Regarding the divergent findings on the correlation between pituitary volume and hormone levels in the two studies, subtle differences in several methodological aspects could be contributing factors. These may include variations in MRI scanning parameters, detailed criteria for pituitary volume delineation, and the sensitivity of hormone detection assays. Furthermore, on a statistical level, the inherent high heterogeneity of the PCOS patient population is a crucial consideration. The study cohorts might have differed in key metabolic characteristics such as the degree of IR, androgen levels, and obesity, which could account for the differing correlations observed. Additionally, the discrepancy might stem from the patients in the two studies representing different disease stages, such as variations in the prevalence of obesity within the PCOS populations, leading to alterations in leptin levels. The association found in the first study could represent a relatively early or active phase of PCOS, where the pituitary gland remains sensitively responsive to aberrant GnRH stimulation, resulting in functional hypertrophy. This phase is more commonly observed in non-obese patients or those without significantly elevated leptin levels, whereby the preserved sensitivity of the hypothalamic-pituitary axis to GnRH leads to excessive LH secretion and potential reactive pituitary enlargement. In contrast, the population in the second study might be in a relatively stable or adapted state, where pituitary enlargement has become a fixed, plateaued manifestation, decoupled from immediate hormonal fluctuations. This stage is likely associated with a higher prevalence of obesity. In obese PCOS patients, elevated leptin levels may exert an inhibitory effect on the hypothalamic GnRH pulse generator, thereby attenuating excessive LH secretion. Such long-term neuroendocrine adaptation may diminish the driving forces for continued pituitary enlargement, resulting in a “plateau phase” or less pronounced morphological changes.

### 3.2 Hypothalamus imaging alterations in patients with PCOS

Current studies on hypothalamic changes in PCOS patients have primarily elucidated on functional and neuroendocrine mechanisms (21). For instance, PCOS is associated with an increased pulse frequency of gonadotropin-releasing hormone (GnRH) and dysregulation of the hypothalamic-pituitary-adrenal (HPA) axis (3, 5). However, research on structural or functional alterations of the hypothalamus in PCOS remains limited.

### 3.3 Cerebral gray matter imaging alterations in patients with PCOS

Gray matter alterations in PCOS are not uniform but demonstrate region-specific patterns, which may underlie some of the associated neuropsychiatric symptoms. In particular, structural changes within brain areas involved in emotional regulation, such as the prefrontal cortex and amygdala, are commonly reported and may be relevant to the mood disturbances frequently observed in patients (22). Furthermore, Gray matter abnormalities in PCOS are associated with dysregulated hypothalamic GnRH secretion, IR, and ovarian-derived sex hormone dysregulation, collectively exacerbating disease progression (23–26).

**Figure 2:**
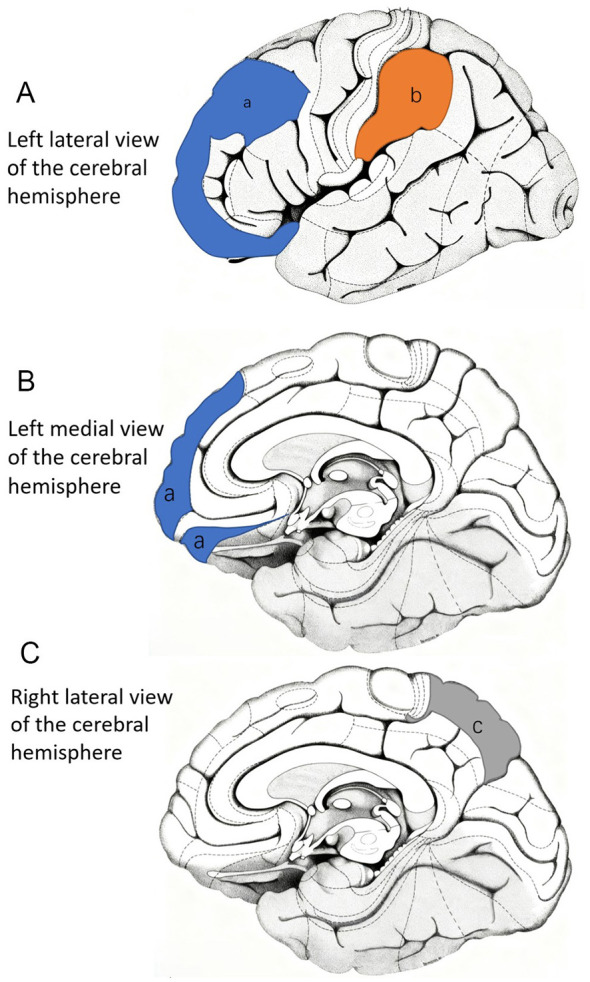
Regional gray matter alterations in patients with PCOS. **(A)** Normal-weight patients with PCOS exhibit volume reductions in the left superior frontal gyrus (a) and the left supramarginal gyrus (b). **(B)** Normal-weight patients with PCOS exhibit volume reductions in the left superior frontal gyrus (a). **(C)** Normal-weight patients with PCOS exhibit volume reductions in the right superior parietal gyrus (c).

Previous studies comparing brain volumes via MRI between normal-weight (BMI 18.5–24.9 kg/m^2^) PCOS patients and healthy controls have demonstrated 10%−17% reductions in frontal and parietal cortical volumes among PCOS individuals. Specifically, the volume of the left superior frontal cortex exhibited a 10% reduction, while decreases of 14% and 10% were observed in the left supramarginal gyrus and the right superior parietal gyrus, respectively (Figure 2). However, no significant differences were found in total intracranial volume, ventricular volume, or cortical thickness between groups (27). Another study enrolled obese (BMI ≥ 30 kg/m^2^) and lean (18.5 < BMI < 25 kg/m^2^) PCOS patients, who were compared with BMI-matched healthy women. All participants underwent brain MRI and measurements of appetite-related hormones, including fasting ghrelin, leptin, and glucagon-like peptide-1 (GLP-1). The results showed that obese PCOS patients exhibited significantly lower total brain volume (TBV) and total gray matter volume (GMV) compared to controls, together with reduced volumes in appetite-related gray matter regions such as the left ventral thalamus, left caudate nucleus, and bilateral hippocampi. In lean PCOS patients, neither TBV nor GMV showed significant differences compared with healthy controls. However, a significant reduction in GMV was observed specifically in the left amygdala (the implications of limbic system structural alterations are discussed in detail in Section 3.4) (12).

Collectively, these findings in primarily young to middle-aged women suggest that GMV reductions are observed across obesity statuses (obese, lean, and normal-weight). However, it remains to be determined whether similar patterns exist in adolescents with PCOS, where the brain is still developing, or in older postmenopausal populations, where age-related and vascular factors may predominate (22, 28, 29). However, it remains inconclusive whether the brain structural changes lead to the emotional and behavioral abnormalities in PCOS patients, or whether chronic emotional stress or hormonal abnormalities cause these structural alterations. This is more likely a bidirectional association rather than a simple causal relationship. The abnormal hormone levels in PCOS patients can directly act on the limbic system, potentially affecting neurotransmitter transmission and inducing microstructural changes in the brain (12, 25, 30). Conversely, chronic anxiety and depression in PCOS patients can lead to persistent activation of the HPA axis, causing abnormal cortisol levels, whose neurotoxic effects may contribute to volume reduction in certain brain regions (31–33).

### 3.4 The limbic system: a central hub for neuropsychiatric symptoms in PCOS

The limbic system, a network of brain regions including the amygdala, hippocampus, anterior cingulate cortex, and nucleus accumbens, is integral to emotion processing, stress response, reward valuation, and memory (34–36). In PCOS, converging evidence from structural, metabolic, functional, and neurochemical imaging points to multifaceted alterations within this system, positioning it as a key neural substrate linking endocrine-metabolic dysfunction to the disorder's neuropsychiatric manifestations (12, 27, 37, 38).

Neuroimaging studies converge on structural and functional alterations within the limbic system of women with PCOS. A focal reduction in gray matter volume is evident in the left amygdala of lean patients, while obese individuals present with more extensive deficits that also encompass the amygdala and hippocampus (12). This structural compromise in core limbic regions establishes a potential anatomical foundation for disturbed neural circuitry. Functionally, a state of brain insulin resistance is reflected in negative correlations between systemic insulin levels and activity in key limbic areas, including the amygdala, hippocampus, and anterior cingulate cortex (37), directly linking metabolic dysregulation to limbic network function. When processing negative emotional stimuli, untreated patients show heightened activation in the bilateral ventral anterior cingulate cortex and left prefrontal cortex (38), indicating a dysregulated limbic-prefrontal response that parallels their clinical mood symptoms. At a neurochemical level, PCOS is associated with elevated μ-opioid receptor availability in the right amygdala and left nucleus accumbens (38). This specific alteration correlates with poorer mood scores, implicating dysregulated endogenous opioid signaling within limbic reward and emotion circuits in the affective pathophysiology of the syndrome.

Collectively, these disparate lines of imaging evidence converge on the limbic system. Its structural compromise, functional dysregulation in response to metabolic and emotional stimuli, and neurochemical imbalance likely forms a unified neural basis for the co-occurring anxiety, depression, and reward-processing changes in PCOS. This perspective underscores the need to investigate PCOS not merely as a peripheral endocrine disorder but as a condition of compromised brain-body communication, with the limbic system at its core.

### 3.5 Cerebral white matter imaging alterations in patients with PCOS

White matter microstructure alterations, particularly those involving connections within and between prefrontal-limbic circuits, are increasingly recognized in psychiatric disorders such as major depressive disorder and bipolar disorder. These subtle white matter changes are implicated in the cognitive deficits and emotional dysregulation that characterize these conditions (39–42). Notably, similar cognitive-affective symptoms are frequently reported in PCOS. This phenomenological overlap, coupled with the established role of insulin resistance and hormonal fluctuations in influencing white matter integrity, strongly suggests that investigating subtle white matter alterations in PCOS is crucial. Such research may reveal a shared neural substrate for affective and cognitive symptoms across these disorders and elucidate the specific brain-body pathways linking endocrine-metabolic dysfunction to neuropsychiatric outcomes in PCOS (43–45).

A DTI study with age- and BMI-matched cohorts (18 PCOS patients vs. 18 healthy controls) revealed no significant differences in overall white matter integrity or myelination between groups. Notably, the PCOS cohort exhibited an elevated tissue volume fraction in the corpus callosum and anterior white matter, demonstrating a neuroanatomical profile that diverges from typical female patterns and trends toward a more male-like phenotype (46). This phenomenon may be attributed to the organizational effects of hyperandrogenism on the nervous system in PCOS patients. Elevated androgen levels potentially alter the construction of neural connections, particularly in commissural fibers such as the corpus callosum (47, 48). Additionally, the inherent IR in PCOS may contribute to these cerebral structural alterations. Insulin plays crucial roles in neuronal survival, synaptic plasticity, and myelination. Consequently, IR in patients may disrupt normal white matter metabolism and maintenance (49–51).

In a cross-sectional MRI study focusing on postmenopausal women with polycystic ovary syndrome (PCOS) under 60 years of age and age-matched healthy controls (±1 year), the PCOS group exhibited a significantly higher prevalence of white matter lesions (40% vs. 22%) and greater severity of subcortical white matter lesions. Furthermore, these patients showed an increased incidence of silent cerebral infarcts. Recent evidence suggests that these findings primarily indicate a significantly increased risk of cerebrovascular disease specifically among obese postmenopausal women with PCOS. These findings collectively indicate an elevated risk of cerebrovascular disease associated with PCOS in the postmenopausal population (52, 53). The increased burden of WMLs and cerebrovascular risk described here is a finding specifically associated with the postmenopausal stage in PCOS, particularly in the context of obesity. It underscores a critical interaction between aging, menopause-related estrogen decline, and lifelong metabolic insults accrued in PCOS. This profile is distinct from the more subtle white matter microstructural alterations reported in younger reproductive-aged women with PCOS (46), highlighting how the neural correlates of PCOS may evolve across the lifespan. Obesity represents a prevalent and pivotal phenotype of PCOS, capable of significantly amplifying the inherent metabolic dysregulation associated with the syndrome, thereby synergistically elevating vascular risk. The increased burden of WMLs and cerebrovascular risk in these individuals may be linked to their underlying metabolic disturbances, which are exacerbated by obesity. IR and hyperinsulinemia can impair vascular endothelial function and promote atherosclerosis, with obesity serving as one of the primary drivers of insulin resistance (54). Additionally, the chronic low-grade inflammation present in PCOS can release inflammatory cytokines that further drive arteriosclerosis and endothelial injury, whereas adipose tissue—particularly visceral fat—serves as a major source of these pro-inflammatory cytokines, thereby exacerbating this inflammatory state (55). After menopause, the protective effect of estrogen diminishes, allowing the negative impact of these metabolic abnormalities to become more pronounced, thereby unmasking the full extent of the synergistic detrimental effects arising from the combination of obesity and PCOS-associated metabolic disturbances (56). These factors collectively exacerbate cerebral small vessel disease, leading to impaired regulatory capacity and increased permeability of small cerebral arteries, ultimately resulting in cerebral white matter ischemia and injury. Therefore, cerebrovascular risk management in postmenopausal women with PCOS should prioritize the obese subgroup, with proactive implementation of lifestyle modifications and targeted metabolic interventions.

## 4 Imaging signatures of glucose metabolic in PCOS patients

**Figure 3:**
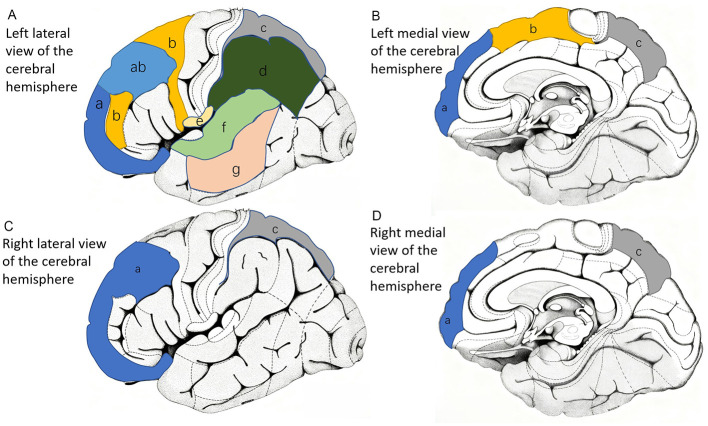
Decreased cerebral glucose metabolism in patients with PCOS. **(A)** Left lateral view of the cerebral hemisphere. Decreased cerebral glucose metabolism in patients with PCOS. **(B)** Left medial view of the cerebral hemisphere. **(C)** Right lateral view of the cerebral hemisphere. **(D)** Right medial view of the cerebral hemisphere. **(a)** Patients with PCOS exhibit decreased glucose metabolism in the superior frontal gyrus (a, ab). **(b)** Left middle frontal gyrus (b, ab). **(c)** Superior parietal lobule. **(d)** Left inferior parietal lobule. **(e)** Left subcentral gyrus. **(f)** Left superior temporal gyrus. **(g)** Left middle temporal gyrus.

Positron emission tomography-computed tomography (PET-CT) enables high-sensitivity detection and dynamic monitoring of cerebral glucose metabolism by imaging radiolabeled glucose, reflecting regional glucose metabolic rates (57, 58). A study controlling for age- and obesity-related IR enrolled young PCOS patients (age: 24.6 ± 5.9 years; BMI: normal range) and age-matched healthy women (age: 24 ± 3.3 years). Quantitative PET-CT analysis revealed significant reductions (9%−13%) in the cerebral glucose uptake of specific frontal, parietal, and temporal cortical regions in the PCOS group compared with controls. These hypometabolic areas encompassed the superior frontal gyrus, left middle frontal gyrus, superior parietal lobule, left inferior parietal lobule, left subcentral gyrus, left superior temporal gyrus, left middle temporal gyrus, hippocampus, and amygdala (Figure 3). Notably, glucose hypometabolism in these regions exhibited significant negative correlations with IR (27). Previous studies in middle-aged/elderly populations and Alzheimer's disease (AD) patients have similarly established links between IR and reduced cerebral glucose uptake in frontal, temporal, and parietal cortices. Therefore, IR may represent a fundamental mechanism underlying the observed hypometabolism in young women with PCOS (59–61).

Research into how IR contributes to glucose dysregulation and affects cerebral energy metabolism in PCOS indicates that impairments in the blood-brain barrier (BBB) and disruptions in neuronal signaling pathways may underlie these cerebral metabolic alterations (25, 62–64). In PCOS patients, IR and chronic low-grade inflammation can damage tight junction proteins between endothelial cells of the BBB, compromising its integrity and allowing harmful substances to enter the brain, thereby disrupting internal homeostasis (65–67). The function of glucose transporters (e.g., GLUT1) on the BBB may also be impaired under IR conditions, hindering stable glucose supply to the brain (68, 69). The disruption of neuronal insulin signaling pathways impairs insulin binding and downstream signaling in neurons, which in turn reduces the transport efficiency of glucose transporter 3 (GLUT3) and compromises mitochondrial function (70, 71). Collectively, these factors contribute to reduced glucose uptake (local cerebral hypometabolism) in specific brain regions of PCOS patients.

IR plays a pivotalrole in the pathogenesis of PCOS. Recent BOLD-fMRI studies identified negative correlations between insulin levels and activity in several brain regions, including key limbic areas, in PCOS patients, a pattern suggestive of brain insulin resistance (see Section 3.4 for a focused discussion on limbic system dysfunction) (37). However, this study has demonstrated suppressed brain responses to food images following glucose ingestion in healthy populations (37). Taken together, these contrasting findings highlight the potential role of brain insulin resistance, particularly within key limbic and prefrontal regions, as a driver of psychopathology (e.g., mood and cognitive disturbances) in PCOS, distinguishing it from normal glucoregulatory neural responses. A study comparing insulin-sensitive and IR PCOS patients utilized fMRI to assess cortical-limbic BOLD responses during high-calorie (HC)/low-calorie (LC) food image viewing after water or glucose ingestion. Significant between-group differences emerged in the DLPFC, amygdala, and fusiform gyrus. Post-glucose HC image viewing elicited positive IR-BOLD correlations in the medial prefrontal cortex (mPFC), OFC, anterior cingulate cortex, and ventral tegmental area (VTA). For LC images, positive correlations were observed in the DLPFC, mPFC, anterior cingulate cortex, and insula. In the insulin-sensitive group, the mean BOLD signal intensity for food images post-glucose ingestion was lower compared to post-water ingestion. Conversely, IR individuals exhibited elevated activity in specific brain regions following glucose intake, whereas insulin-sensitive individuals demonstrated suppressed responses (72). This phenomenon may be attributed to impaired appetite regulation in patients with PCOS. In PCOS patients with the IR subtype, insulin may fail to effectively signal the brain's reward system to inhibit responses, resulting in a failure to suppress neural excitability toward food cues post-glucose intake (73). Consequently, following glucose intake, patients exhibited increased activity in specific brain regions, whereas a decrease was observed in the insulin-sensitive subtype. Furthermore, IR might disrupt the functional integrity of the mesolimbic dopamine system by specifically altering dopamine release in the ventral tegmental area (VTA) and nucleus accumbens (NAc), thereby leading to dysregulation of the reward circuitry (73–75). Beyond the failure in regulatory systems, patients with PCOS may also experience impaired cerebral energy utilization due to IR, which could conversely promote regional brain activation through a negative feedback mechanism (73, 76).

## 5 Imaging correlates of disordered eating behaviors in patients with PCOS

**Figure 4:**
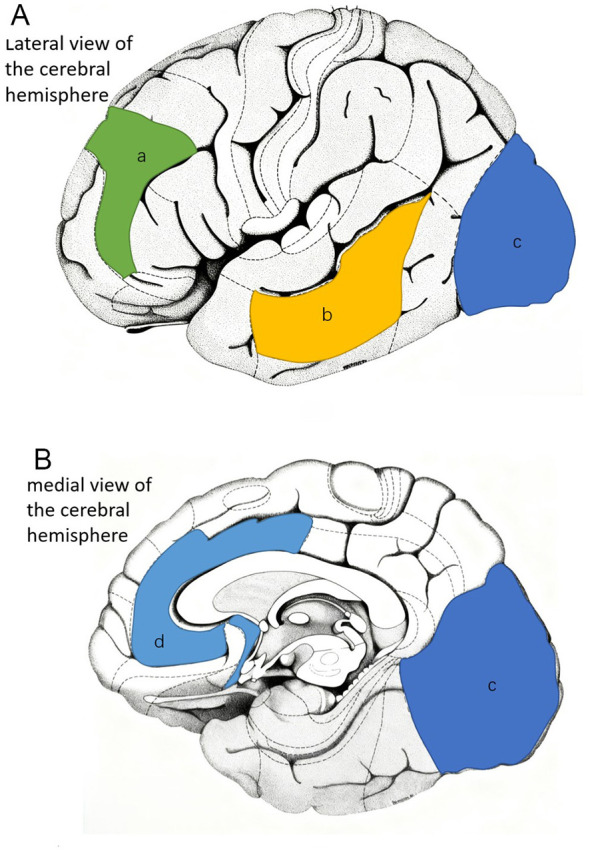
Brain regions associated with eating disorders in patients with PCOS. **(A)** Lateral view of the cerebral hemisphere. **(B)** medial view of the cerebral hemisphere. **(a)** Insular cortex. **(b)** Visual cortex. **(c)** Middle temporal gyrus. **(d)** Anterior cingulate cortex.

PCOS patients frequently exhibit various eating disorders, such as binge eating, selective hyperphagia (characterized by a preferential intake of high-fat, high-salt, or high-sugar foods), and restrictive eating (involving severely reduced caloric intake). These disordered eating behaviors and the underlying endocrine dysfunction and IR can exacerbate each other, forming a vicious cycle through mechanisms related to dysregulated nutrient partitioning (77, 78). Neuroimaging studies have identified several brain regions implicated in the regulation of hunger and satiety in this context. These regions include the dorsomedial and dorsolateral prefrontal cortex (DMPFC/DLPFC), OFC, anterior cingulate cortex, middle temporal gyrus, visual and insular cortices, thalamus, amygdala, hypothalamus, hippocampus, midbrain, striatum, and cerebellum (Figure 4) (79).

A retrospective study comparing the apparent diffusion coefficient values in various brain regions and nuclei between normal-weight women with PCOS and healthy controls revealed significantly elevated apparent diffusion coefficient values in the PCOS group. These increases were observed in several regions, including the thalamus, insular cortex, visual cortex, middle temporal gyrus, anterior cingulate cortex, and DLPFC. This pattern of elevation suggests the presence of microstructural alterations and possible cellular volume loss in these cerebral areas of PCOS patients (80). Structural alterations in the visual occipital cortex may heighten its sensitivity to food-related visual cues, potentially driving intense cravings and disordered eating behaviors (81, 82). Furthermore, these structural changes could disrupt functional coordination between the occipital cortex and other brain regions involved in interoceptive processing, such as the insula. Specifically, hyperreactivity of the visual cortex to food cues may override insular satiety signaling, leading to impaired satiety perception or aberrant hunger signaling. Consequently, affected individuals may continue eating despite sufficient caloric intake, thereby exacerbating binge eating behaviors (83–85). Separately, structural alterations in the DLPFC may disrupt the accurate reception and integration of gastrointestinal satiety hormone signals (86, 87). This impairment can lead to the premature suppression of feeding motivation, potentially contributing to dysregulated eating behaviors (79). These region alterations may collectively underlie aberrant eating behaviors in PCOS.

## 6 Cortical alteration associated with cognitive dysfunction in PCOS

Cognitive function encompasses the brain's ability to process, store, and retrieve information, which constitutes the fundamental psychological basis for human activities (88, 89). Neuroimaging and neuropsychological studies have established that the parietal, temporal, and occipital lobes are critically involved in attentional allocation, language comprehension, memory formation, and cognitive integration (90–93). Compared with healthy individuals, PCOS patients frequently exhibit impairments in multiple cognitive domains, including visuospatial ability, verbal fluency, and information processing speed. These deficits are particularly pronounced in tasks demanding verbal memory and working memory (44, 94, 95).

**Figure 5:**
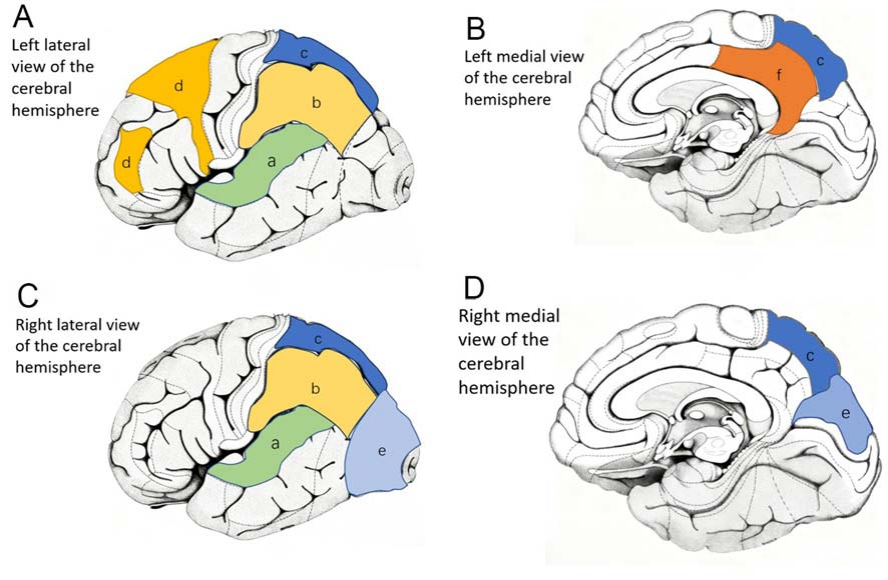
Brain regions associated with highly activated cognitive areas in patients with PCOS. **(A)** Left lateral view of the cerebral hemisphere. **(B)** Left medial view of the cerebral hemisphere. **(C)** Right lateral view of the cerebral hemisphere. **(D)** Right medial view of the cerebral hemisphere. **(a)** Superior temporal gyrus. **(b)** Superior parietal gyrus. **(c)** Inferior parietal gyrus. **(d)** Left middle frontal gyrus. **(e)** Left posterior cingulate gyrus. **(f)** Right middle occipital gyrus.

A working memory neuroimaging study comparing PCOS patients and healthy controls demonstrated bilateral activation in task-related regions, including the DLPFC, insula, parietal lobe, cerebellum, and superior medial prefrontal cortex. Furthermore, these untreated patients exhibited heightened activation in cognition-related regions (e.g., the superior temporal gyrus and superior/inferior parietal gyri) compared to controls, with the most pronounced differences observed in parietal activation (Figure 5). Such overactivation in brain regions may represent a compensatory mechanism during the execution of cognitive tasks: in PCOS patients, metabolic factors such as IR can lead to reduced functional activity in specific brain regions. This impairment compromises the neurons' ability to effectively utilize glucose as an energy source and diminishes the neurotrophic and protective functions of insulin. Consequently, synaptic plasticity and the efficiency of neural signaling may be adversely affected, leading to inefficient brain processing (96–99). To compensate for this inefficiency, the brain may need to recruit broader neural regions and operate at a higher intensity to accomplish cognitive tasks. Furthermore, hyperandrogenism in PCOS can directly affect brain regions rich in androgen receptors, such as the prefrontal cortex and hippocampus, potentially interfering with normal neurotransmission and network connectivity (25, 48). The observation that this overactivation diminishes and task accuracy improves following anti-androgen treatment supports the plausibility of this hypothesis, suggesting that androgen regulation may ameliorate PCOS-related cognitive deficits (27).

**Figure 6:**
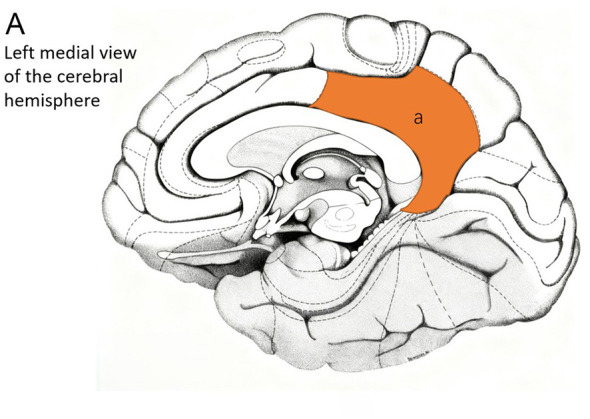
Brain regions associated with cognitive impairment in patients with PCOS. **(A)** Left medial view of the cerebral hemisphere. **(a)** Left posterior cingulate gyrus.

Another study on functional brain activity enrolled age- and education-matched PCOS patients and healthy women. Results revealed reduced ALFF in the left middle frontal gyrus (MFG.L), left posterior cingulate gyrus (PCG.L), and right middle occipital gyrus (MOG.R) in PCOS patients. Concurrently, decreased FC strength between the right inferior occipital gyrus (IOG.R) and MOG.R was positively correlated with working memory task accuracy, suggesting that hypoactivity in MOG.R and IOG.R may underlie working memory deficits in PCOS (Figure 6). The study further identified enhanced FC strength between MFG.L and the left inferior frontal gyrus (IFG.L), which correlated positively with testosterone (T) levels. Increased activity in MFG.L and IFG.L also showed positive correlations with T, aligning with prior evidence implicating androgen excess in cognitive dysfunction (22, 100). Nevertheless, inconsistent findings regarding the associations between cognitive performance and T or insulin AUC in PCOS have been reported, thus requiring further investigation (46).

Additionally, MFG.L ALFF exhibited a negative correlation with fasting insulin levels in IR PCOS patients. This correlation was not observed in non-IR individuals (22). This implies that structural changes in the MFG.L may arise from IR, leading to a correlation between its neural activity and circulating insulin levels. Moreover, researchers believe that the alterations in the MFG.L are the cause of cognitive impairment in PCOS patients.

## 7 The cerebral cortex changes underlying affective disturbances in PCOS

Cortical regions, including the prefrontal cortex, insula, and anterior cingulate cortex, as well as key subcortical and limbic structures such as the amygdala, hippocampus, and nucleus accumbens, are integral to the neural circuitry governing emotional regulation. Previous studies report enhanced activation in the right nucleus accumbens, bilateral amygdala, prefrontal cortex, and anterior cingulate cortex, coupled with reduced activation in the PCG.L in depressed patients. PCG.L plays a pivotal role in emotional circuitry and is strongly associated with depressive symptoms (101–103). PCOS patients frequently present with clinical features such as hirsutism, obesity, and acne. These physical manifestations often lead to body image dissatisfaction. Furthermore, concurrent infertility and prolonged treatment regimens exacerbate psychosocial stress, which may interact with the underlying hormonal and metabolic dysregulation in PCOS to increase the risk or severity of anxiety and depression (4, 104–106).

**Figure 7:**
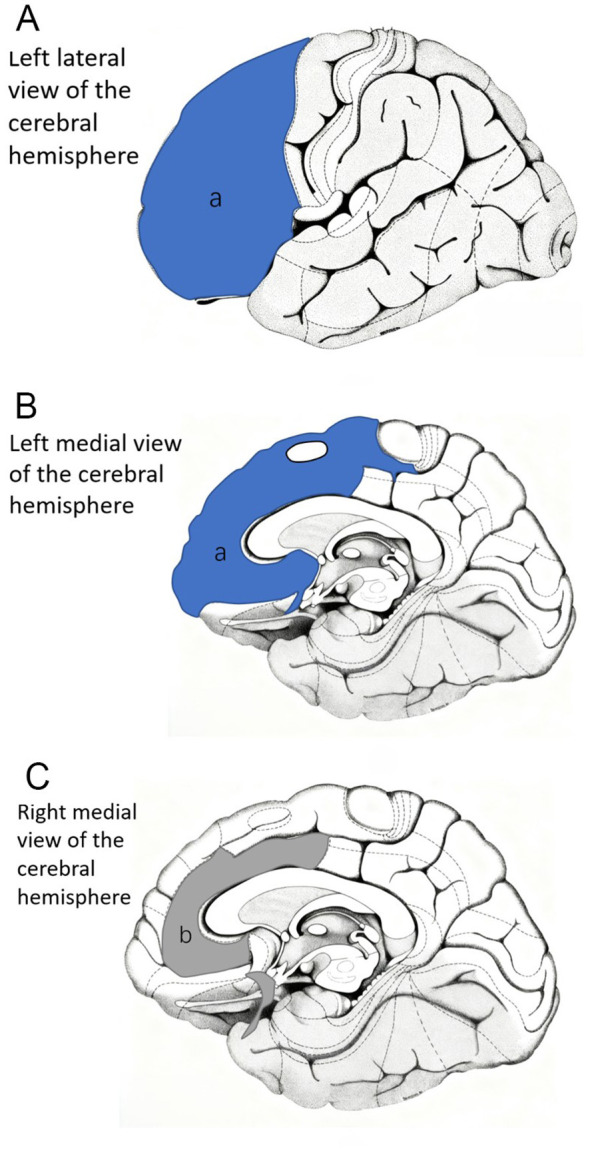
Brain regions activated by negative emotions in patients with PCOS. **(A)** Left lateral view of the cerebral hemisphere. **(B)** Left medial view of the cerebral hemisphere. **(C)** Right medial view of the cerebral hemisphere. **(a)** Left prefrontal cortex. **(b)** Left ventral anterior cingulate cortex.

As noted earlier, PCOS patients demonstrate reduced PCG.L ALFF and significantly higher emotional/depression scale scores compared to healthy controls (22). A separate study evaluating emotional processing in PCOS patients vs. controls included fMRI and PET scans during emotion-recognition tasks and anxiety assessments, conducted pre- and post-metformin therapy (38). Untreated PCOS patients exhibited heightened neural activation in the left prefrontal cortex and bilateral ventral anterior cingulate cortex (ACC) upon exposure to negative emotional stimuli (Figure 7), suggesting altered limbic-prefrontal circuitry during affective processing (further elaborated in Section 3.4) (38).

## 8 Brain imaging alterations of sympathetic hyperactivation in PCOS

The sympathetic nervous system (SNS) plays a critical role in both short- and long-term regulation of arterial blood pressure. Upon activation, norepinephrine released from sympathetic nerve endings binds to adrenoceptors in the myocardium, increasing heart rate and enhancing cardiac contractility. Concurrently, SNS-induced vasoconstriction in the peripheral arteries elevates systemic vascular resistance, thereby raising blood pressure. PCOS patients frequently exhibit chronic sympathetic hyperactivity, which is associated with complications such as obesity, hyperinsulinemia, and obstructive sleep apnea (107, 108). To investigate alterations in brain activity under SNS activation in PCOS, a study utilizing the isometric forearm contraction (IFC) model compared age- and BMI-matched PCOS patients to healthy controls. After maximal grip strength was measured, BOLD-fMRI scans and SNS activity assessments were conducted at 30% of maximal voluntary contraction. The results demonstrated that the PCOS group exhibited heightened SNS excitability, along with elevated BOLD signal intensity in the right OFC. This elevated signal correlated positively with the homeostatic model assessment of IR (109). Previous studies identify the right OFC as a key regulator of blood pressure. Thus, improving IR in PCOS may downregulate right OFC activity and attenuate SNS enhanced activation, potentially ameliorating long-term cardiovascular outcomes (e.g., heart failure and coronary artery disease) (110, 111).

## 9 Brain imaging alterations and μ-opioid system in PCOS patients

The opioid system exerts profound effects on behavior, appetite regulation, thermoregulation, respiratory activity, sleep-wake cycle modulation, emotion, and cognition. Endogenous opioid peptides and opioid receptors are central components of this system. Endogenous opioids play critical roles in reproductive neuroendocrine functions, stress responses, and analgesia. The μ-opioid receptor, a key receptor subtype, is widely distributed throughout the central nervous system, including the cerebral cortex and limbic system, and is implicated in reducing pain sensitivity and emotional distress (112–114). Previous studies identified altered μ-opioid receptor-mediated neurotransmission in depressed women compared to healthy controls. Specifically, heightened opioid system activation during emotional challenges was observed in depression/anxiety-associated regions such as the right nucleus accumbens and bilateral amygdala (101). The amygdala and nucleus accumbens, integral components of the limbic system, are closely involved in pain signal transmission, emotional processing, and autonomic regulation (115–117).

A recent study examining the relationship between emotional processing and the μ-opioid receptor system in PCOS patients assessed emotional and anxiety status using standardized scales and employed PET imaging to evaluate μ-opioid receptor availability (density/affinity) during emotional tasks. The results demonstrated elevated μ-opioid receptor availability in the right amygdala and left nucleus accumbens of the PCOS cohort compared with healthy controls. These elevations were positively correlated with negative mood and depression scores (38). This phenomenon may be attributed to the chronic stress and emotional distress frequently experienced by women with PCOS, which leads to the excessive release and subsequent depletion of endogenous opioid peptides, such as β-endorphin (118–120). To maintain system homeostasis, neurons might upregulate receptor expression to enhance signal detection capability. Alternatively, the underlying endocrine disturbances in PCOS, such as hyperandrogenism, could directly alter the receptor expression profile, given that androgens are known to modulate the expression of opioid receptors within the central nervous system (121, 122).

### 9.1 Summary and prospect

This review comprehensively explores neuroimaging and functional alterations in patients with PCOS, revealing their close associations with endocrine-metabolic disturbances, behavioral changes, cognitive deficits, and emotional dysregulation. Significant imaging alterations in PCOS patients include: enlarged pituitary volume correlated with elevated LH levels and LH/FSH ratios (see studies in Table 1); reduced gray matter volumes, particularly in the frontal and parietal lobes and amygdala, potentially linked to mood disorders and cognitive dysfunction (see studies in Table 1); dysfunction of the limbic system (encompassing structural, functional, and neurochemical changes in regions like the amygdala and hippocampus) (see studies in Table 1); the risk of white matter abnormalities is increased, which is also linked to underlying cardiometabolic risk factors (see studies in Table 1). Additionally, glucose metabolism abnormalities in PCOS patients are associated with reduced cerebral glucose uptake, while the severity of IR correlates with altered activity in specific brain regions (see studies in Table 1). Regarding eating behaviors, microstructural alterations in the cerebral cortex of PCOS patients may lead to disordered eating patterns. In terms of cognitive function, PCOS patients exhibit abnormal activation in brain regions during working memory and other cognitive tasks. For emotional regulation, mood disturbances in PCOS are associated with functional abnormalities in specific brain areas. Finally, PCOS patients demonstrate SNS activation associated with IR and alterations in the activity of the limbic μ-opioid receptor system (see Table 1).

Neuroimaging can help researchers and clinicians better understand the pathological changes and related comorbidities of PCOS. However, the number of neuroimaging studies on the brain in PCOS patients is currently limited, and findings regarding altered brain regions are inconclusive, primarily demonstrating correlations rather than causation. There is a particular lack of systematic research and synthesis on the distinctions among various PCOS subtypes and how brain regions are altered in each subtype. Key questions, such as whether brain alterations are the cause or consequence of PCOS, whether there is a direct relationship between these brain changes and the various symptoms exhibited by PCOS patients, and the underlying mechanisms involved, need to be addressed. Critically, these questions must be explored within the context of distinct PCOS phenotypes (e.g., metabolic, ovarian, adrenal, or mixed subtypes). Furthermore, a life-course perspective is essential. PCOS is not static, and its interplay with the brain evolves from adolescence through menopause. The current synthesis primarily reflects findings in reproductive-aged women. Future research must explicitly address how brain alterations differ across key life stages. For instance, are the neural changes observed in adolescents with PCOS predictive of long-term outcomes or do they represent a transient, development-related state? How do the neurodegenerative and cerebrovascular risks identified in postmenopausal populations originate from earlier-life neural and metabolic dysregulation? Longitudinal studies tracking the same individuals from adolescence into later adulthood are desperately needed to disentangle cause from consequence, and to identify critical windows for intervention. A fundamental step forward lies in adopting a phenotype-specific framework. Future studies must integrate deep metabolic and hormonal phenotyping with multimodal neuroimaging in large cohorts to dissect the unique brain-body pathways operative in each subgroup. This approach shifts the core question from a broad “cause or effect” to a more precise inquiry: what is the nature and direction of the interaction between specific neural circuit dysfunctions and the distinct pathophysiologies of each phenotype? For instance, in the metabolic phenotype, the priority is to discern whether brain insulin resistance within reward and cognitive circuits initiates hyperphagia and mood dysregulation—thereby worsening peripheral insulin resistance—or whether it emerges as a downstream effect of sustained hyperinsulinemia. Research on the ovarian phenotype? should clarify if the disordered hypothalamic-pituitary-ovarian axis feedback stems primarily from intrinsic hypothalamic dysregulation or is secondary to impaired higher-order limbic inputs that disrupt GnRH pulsatility. In the adrenal phenotype, key work will involve establishing whether heightened activity in stress-processing regions like the amygdala and prefrontal cortex precedes and promotes adrenal hyperandrogenism via the HPA axis, or constitutes a consequence of it. Elucidating these distinct pathways is crucial. It will advance the field from observing correlations to defining mechanisms, ultimately testing the hypothesis that PCOS represents, in significant part, a disorder of brain-body communication whose clinical face varies fundamentally across these phenotypic subgroups. Future research should combine neuroimaging with the pathophysiology of PCOS in larger cohorts to elucidate the brain imaging alterations and specific mechanisms across different PCOS phenotypes.

## Glossary

**Abbreviations:** PCOS, Polycystic ovary syndrome; HPO, hypothalamic-pituitary-ovarian; FSH, follicle-stimulating hormone; LH, luteinizing hormone; MRI, Magnetic resonance imaging; BMI, body mass index; PGV, pituitary gland volume; GnRH, gonadotropin-releasing hormone; HPA, hypothalamic-pituitary-adrena; IR, insulin resistance; GLP-1, glucagon-like peptide-1; TBV, total brain volume; GMV, gray matter volume; DTI, Diffusion tensor imaging; PET-CT, Positron emission tomography-computed tomography; AD, Alzheimer's disease; DLPFC, dorsolateral prefrontal cortex; OFC, orbitofrontal cortex; AUC, area under the curve; HC, high-calorie; LC, low-calorie; mPFC, medial prefrontal cortex; VTA, ventral tegmental area; DMPFC, dorsomedial/dorsolateral prefrontal cortex; ALFF, amplitude of low-frequency fluctuation; MFG.L, left middle frontal gyrus; PCG.L, left posterior cingulate gyrus; MOG.R, right middle occipital gyrus; IOG.R, right inferior occipital gyrus; IFG.L, left inferior frontal gyrus; T, testosterone; SNS, Sympathetic nervous system; IFC, isometric forearm contraction; WMLs, white matter lesions.
